# Did Australian policy prepare for a harmful algal bloom with significant human health impacts? Analysis and lessons from South Australia

**DOI:** 10.1093/heapro/daaf240

**Published:** 2026-01-21

**Authors:** Frances Baum, Matthew Fisher, Diana Bogueva, Amanda Hayes, Helen Miller, Dora Marinova

**Affiliations:** Stretton Health Equity, School of Public Health, Adelaide University, North Terrace, Adelaide, South Australia 5000, Australia; Stretton Health Equity, School of Public Health, Adelaide University, North Terrace, Adelaide, South Australia 5000, Australia; Curtin University Sustainability Policy Institute, Curtin University, GPO Box U1987, Perth Western Australia 6845, Australia; Stretton Health Equity, School of Public Health, Adelaide University, North Terrace, Adelaide, South Australia 5000, Australia; Curtin University Sustainability Policy Institute, Curtin University, GPO Box U1987, Perth Western Australia 6845, Australia; Curtin University Sustainability Policy Institute, Curtin University, GPO Box U1987, Perth Western Australia 6845, Australia

**Keywords:** Harmful Algal Bloom, One Health, respiratory health, mental health, healthy public policy, climate change, intersectoral action, sustainability

## Abstract

A harmful algal bloom (HAB) has spread to a third of South Australia’s coasts, has devastated marine life and is affecting human physical and mental health and the fishing and tourism industries. We examine Australian Federal and State environmental and agricultural public policies to determine how well they were prepared to prevent and respond to a HAB with human health consequences. Document analysis techniques were used to examine the framing of 63 Australian public policies selected from a data set of 180. All documents were coded in Nvivo and analysed for their content relevant to HABs. In the policies, we only found cursory mentions of HABs. We identified three main issues in terms of their attention to HABs. First, there was no evidence of policies that would have led to any detailed planning for a HAB. Second, in terms of strategic actions, economic considerations are uppermost. Third, we found little consideration of direct human health impacts or of intersectoral collaboration on the issue. Our policy analysis indicates a lack of attention, and so preparedness, for the South Australia HAB. Public policy in all countries needs to prepare better for climate-related disasters and act faster to reduce global warming.

Contribution to Health Promotion

- Assessment of the extent to which Australian governments’ policies were prepared for Harmful Algal Blooms (HABs) of the type experienced from March 2025 in South Australia
- Identification of awareness of threats to human health from HABs
- Discussion of what healthy public policy in relation to a HAB would look like
- Examination of a health threat from global warming and how policy could help shape response
- Value of intersectoral healthy public policy in responding to ecological disasters and their human health impacts

## Introduction

In mid-March 2025, a harmful algal bloom (HAB), caused primarily by the dinoflagellates Karenia Mikimotoi and Karenia Cristata was first detected on South Australian Fleurieu Peninsula beaches. It has since expanded to over ∼4400–4500 km^2^ across the state’s coastal waters and penetrated delicate wetlands (Government of South Australia 2025a). According to Glibert *et al*. (2001), HABs occur when marine algae proliferate to levels that damage ecosystems causing fish and shellfish mortality and multiple human health impacts. The bloom has caused catastrophic marine mortality—impacting 400+ species, including fish, rays, sharks, sea dragons, cuttlefish, shellfish, seabirds and dolphins (Government of South Australia 2025a, Great Southern Reef Foundation 2025, Tian 2025). The causes of the bloom have been attributed to three main factors: a prolonged marine heatwave since September 2024, raising sea temperatures by ∼2.5°C above normal; nutrient influx from the 2022–23 Murray River floods and cold-water upwelling events (Government of South Australia 2025a). Calm seas and intense sunlight further fuelled its persistence. The bloom expanded at ∼2.5 km per day, behaving like a ‘moving beast’, may persist for up to a year unless disrupted by significant weather shifts, and could affect marine life for 5 years (Baker 2025, Gratton 2025). While the algae is not believed to be directly toxic to humans, it emits reactive oxygen species that cause respiratory, eye, and skin irritation. Health advisories have been issued by the South Australian Department of Health (SA Health), including warnings for residents to keep windows closed during the bloom with particular caution for people with chronic lung diseases (SA Health 2025). Fisheries, aquaculture (including oyster farms), tourism, and local businesses are severely impacted—fishers report near-total income loss, oyster farms have closed, and beaches are littered with dead marine life (Lance 2025). Community members experience shock, sadness, and disbelief at the scale of the disaster reflecting eco-distress, solastalgia (Albrecht *et al*. 2007), and ecological grief, linked to environmental loss (Senate Standing Committees on Environment and Communications 2025a, 2025b).(Chapter 6: Health Impacts of the Algal Bloom).

While scientists have described the 2025 South Australian HAB as ‘one of the worst marine disasters in living memory’ (Government of South Australia 2025a), it is not without precedent. Australia has previously experienced significant blooms causing widespread fish kills and aquaculture losses (Ruth 2014). Internationally, China’s Fujian Province in 2012 suffered major aquaculture losses from Karenia mikimotoi blooms while Japan has documented recurring Karenia mikimotoi blooms since the 1930s, with intensification in recent decades (Gu *et al*. 2022), Similarly, Florida’s Karenia brevis red tides have repeatedly harmed ecosystems, tourism, and human health (Bechard and Lang 2024), costing an estimated $2.7 billion in 2018 alone (Alvarez *et al*. 2024). These examples demonstrate that HABs are not single, black swan events but foreseeable risks, increasingly linked to climate change and nutrient loading. Toxic dinoflagellate blooms, known since the 1930s (Brown *et al*. 2020), have increased in recent decades (Furuya *et al*. 2018) and are recognized as a biosecurity risk likely to intensify with climate change (Brown *et al*. 2020). They have also been associated with health issues and their long-term health effects although recognized, are under-researched and largely unknown (Lim *et al*. 2023). Thus, they could reasonably have been expected to be anticipated in public policies addressing climate change, biosecurity, and aquaculture.

The current event provides a serious climate signal with continuing ecological, health, and economic consequences for South Australia. Our broader research has been considering the extent to which Australia’s environment and agricultural policies are supportive of a healthy and sustainable food supply and in this article, we draw on our policy analysis to consider which Australian policies were prepared to prevent and respond to a HAB with human health consequences.

## Materials and methods

We used standard document analysis techniques to analyse Australian environment, agriculture and food-related policies, which involved collecting, coding, synthesizing and theorizing the research data. Morgan (2022) notes that document analysis is underused in qualitative research even though it can allow a powerful lens to understanding policy historical evolution, actors’ framing, and embedded values. Documents study allows a critical interpretive analysis (van Hulst *et al*. 2025)—key to understanding policy priorities, framing, and focus. We collected all current strategic policies, selected legislative documents and the most recent annual reports from the websites of departments responsible for environment, agriculture and food-related policies outside the health sector in the Federal, all state and territory governments current in 2024–25 which included policies dating back up to 10 years. Each policy was then assessed to ascertain whether it was primarily strategic. We defined strategic policy as including the goals, objectives and strategies of the department regarding a specific area of policy responsibility. Our broader project identified 180 policies in the agriculture and environment sectors that were relevant to the food system. We then text searched these documents to determine whether they mentioned ‘fish’, ‘marine’, or ‘aqua’. If mentions were fewer than four, FB/MF reviewed them for relevance. This process identified 63 policies related to marine, aquaculture and fisheries issues (see Supplementary File for list of policies).

All documents were coded thematically using Nvivo Version 12. Our coding system was adapted from earlier research which has examined policy for its health, equity, and sustainability content (Fisher *et al*. 2014). We added food, agriculture, and environment codes to the existing framework and also codes in relation to evidence of corporate involvement in policy formulation and substance. These codes were then used to analyse the selected policies to determine whether and how the policies aligned with the intent of dealing with the prevention, impact, and mitigation of the algal bloom disaster. Our analysis involved close reading and data familiarization, thematic coding, and identifying emergent framings. Framing analysis provided insights into stakeholders’ values and interests, showing whose perspectives shape policy decisions (Bacchi 1999).

### Ethical approval

The paper is based on analysis of publicly available policy documents and no ethics approval is required.

## Results

In the Australian federated system, responsibility for agriculture, fisheries and environment policy is divided between Federal Government and States—New South Wales (NSW), Queensland, South Australia, Tasmania, Victoria and Western Australia (WA), and Territories—Australian Capital Territory and Northern Territory (Australian Fisheries Management Authority 2023). Federal regulators are responsible for commercial fisheries from three nautical miles out to the extent of the Australian Fishing Zone. The States and the Northern Territory look after recreational, traditional indigenous, commercial, coastal, and inland fishing and aquaculture. Federal and State and Territory Governments make policies relevant to the environment and climate change. In our total set of 180 policies, we only found cursory mentions of marine HABs.

Our review of the agriculture, environment and climate change policies relevant to the HAB indicated three main issues. First, that there was no evidence of policies that would have led to any detailed planning for an algal bloom of the size and duration of that in South Australia in 2025. Second, that while the policies do frequently mention the need for sustainability and preservation of the environment in their background, in terms of strategic actions, economic considerations are uppermost, and third, we found very little consideration of direct human health impacts or of intersectoral collaboration on the issue. These points are expanded on below.

### Lack of attention to harmful algal blooms

We found no policy framework for dealing with an HAB crisis of the nature of the South Australian one. Out of 180 policies, we found only 9 (5%) references to algae and of these only 3 (less than 2%) policies refer to the impact of HABs. The NSW Food Authority (NSW Food Authority 2022) discussed their work to develop a ‘PCR [polymer chain reaction] based assay to detect the presence of toxic algae’ and noted if successful ‘this work could result in a step change in how this risk is managed’.

Similarly, the Victorian Department of Environment, Land, Water and Planning (Department of Environment Land Water and Planning 2022) discussed the fact that: ‘Decreased water quality may include high turbidity and water-borne health risks (such as the presence of harmful algae and pathogenic microorganisms)’. The WA Department of Primary Industries and Regional Development (Department of Primary Industries and Regional Development 2022) required ‘routine sampling and analysis of seawater and oyster flesh to detect toxic algae levels, before any product is to be harvested for domestic and, or, export markets’. While the NSW Department of Primary Industries (2024) policy on climate research might be expected to deal in some detail with the risk of a HAB; no mention is made of ‘algae’ or ‘algal’. It does however have a ‘marine industries’ section and presents detailed assessments of projected impacts on climate change on fisheries related to five ‘key’ species of fish, but this is even though, according to the report:

The most common climate variables used in the marine fisheries models were **sea surface temperature**, sea surface height and ocean current strength …[and] seafloor depth. (p. 86)

Perhaps most strikingly, the South Australian key plan on climate change science and knowledge (Department for Environment and Water 2022) noted: ‘Sea surface temperature variations affect marine ecosystems in a range of ways, including changes in species range, spread of invasive species and changes to disease risks’ (p. 39). The policy however did not mention the risk of HABs to the SA marine environment.

The SA policies relating to climate change did recognize the threats posed in general terms:

South Australia is becoming hotter and drier, with rising sea levels and an increased risk of more frequent and intense heatwaves, bushfires, storms, and floods. (p. 2)

Climate change is already having a direct impact on native wildlife, ecosystems and habitats, and these effects will increase in the future, driving changes in the distribution of species and the ecosystems and habitats they live in.(Department for Environment and Water 2022) (p. 16)

However, these policies did not contain warnings of the impacts of a large scale HAB as seen in 2025. A further document setting out climate actions (South Australian Government 2022) has a section on ‘Agriculture, Landscapes and Habitats’. One of its four strategies is: ‘Build the climate resilience of landscapes, habitats and natural resources’; yet none of the twelve actions areas relate to the threat of HABs.

We found agriculture policies in all jurisdictions did place considerable emphasis on the need for biosecurity protection. A typical statement is:

It has never been more critical to ensure we strategically invest in our biosecurity and food safety systems, not only as we seek to drive Australia’s *primary industries output to $100 billion ($30 billion in NSW) by 2030* but also as we confront a rapidly growing global threat profile. (Department of Primary Industries 2022) p. 2

Despite its focus on biosecurity, this NSW policy did not mention HABs at all. In Queensland, an Annual Report (Department of Agriculture and Fisheries 2023) noted the importance of: ‘Strengthened biosecurity protocols to prevent aquatic pest incursions’, but made no mention of HABs or a strategic framework to deal with any incursion. In WA, a climate change policy (Department of Water and Environmental Regulation 2023) did identify impact to the marine environment as a priority, noting the more frequent marine heatwaves causing coral bleaching, loss of seagrasses and fish deaths, but HABs are not identified.

Thus, despite every state and territory being concerned about the biosecurity risks to agriculture and aquaculture, the risks were mainly conceived in terms of animal or plant diseases or introduced pests with a focus on how this affects primary industries while HABs were simply not named. Given the potential impact of HABs on the aquaculture industries (Lenzen *et al*. 2021), this lack of policy attention is worrying. The policies about the growing aquaculture industries in Australia might have been where most reference to the risks of HABs would be expected but very little was said about this risk. The Ministerial introduction to the salmon industry plan said:

I am pleased to share the Tasmanian Government’s Salmon Industry Plan 2023 (the Plan) which highlights the Government’s commitment to a thriving salmon industry – one that is economically successful, environmentally responsible, socially beneficial and well-managed. (Department of Natural Resources and Environment 2023) (p. 4)

This is despite evidence that aquaculture industries may affect and be affected by HABs (Lenzen *et al*. 2021, Rolton *et al*. 2022). The climate policies show very little consideration of the marine environment. For example, South Australia has a ‘Landscape Strategy’ (South Australian Landscape Boards 2021) but none for the marine environment that is not focused on the needs of the fishing industry. While the Landscape Strategy says in its introductory page it considers: ‘The natural environment including our rivers and plains, forests and hills, coasts and seas, as well as the built environment’, the marine environment is barely mentioned.

### Dominance of economic framing in the policies

We found that the 63 policies we identified as those that might have been expected to provide guidance on a HAB all had a strong economic orientation. Of the 63 policies reviewed, over 90% framed environmental protection primarily in terms of economic growth or market access, with fewer than 5% giving substantive attention to ecological or human health risks. Table 1 provides examples of those orientations. The seafood industry growth strategy in South Australia (Government of South Australia 2021) demonstrates a focus on: ‘Driving growth and development through new market opportunities, products and experiences’ (p. 12) with little regard to either environmental risks or how they might be dealt with. Western Australia’s development plan (Department of Primary Industries and Regional Development 2020) similarly framed environmental protection as a trade advantage:

Competition will also increase in the more discerning middle-class consumer markets as new producers improve their biosecurity, animal welfare, and sustainability credentials.

WA is free of many significant pest and diseases which supports and enables trade and market access. (p. 17)

There are no parts of this WA policy which valued the natural environment and marine habitat as a good in its own right. A common framing in many policies was that the protection of the environment was seen as important to increasing markets for marine products with a particular focus on export markets. The Federal Government’s Delivering Ag 2030 exemplified this framing with its first theme on Trade and Exports and a heading ‘Getting our produce to the world’ (p. 6). The NT Department of Industry, Tourism and Trade Agribusiness 2030 (Department of Industry Tourism and Trade) (Northern Territory Department of Agriculture and Fisheries 2016) highlighted the primary concern with economic growth over considerations of biosecurity and health as is shown in this intended outcome:

Biosecurity resources are clearly planned and focused on areas of greatest return on investment. (p. 15).

In terms of the policies that were directly related to the fishing industry, the key foci were on the sustainability and growth of the fishing industry, biosecurity risks, support for Indigenous traditional fishing practices, and protection from illegal fishing. However, there was no real sign that the Indigenous knowledges were being taken seriously or being used to inform policies in other than rhetorical terms. The overall tenor of the policies was about the need to increase the profitability and revenue from fishing. While most of the detailed strategies we found in the policies concerned economic measures to support domestic and export industries, the framing in the documents did include consideration of ecological sustainability as some of the extracts in Table 1 illustrate. This concern did, however, not follow through to clear measures to protect the environment or prepare for the action required when an ecological disaster of the nature of the South Australian HAB occurs. The Federal plan for fisheries and agriculture (Australian Fisheries Management Authority 2023) has as its first objective ‘Support industry to grow towards a $100 billion agriculture, fisheries and forestry industry by 2030 amid changing global market conditions’ (p. 3) and the second objective (relating to biosecurity) and the third (relating to resilience and sustainability) are still primarily focused on the needs of industry rather the environment.

**Table 1:** Examples of industry orientation of Australian fisheries policy.

Policy	Extract from policy
Australian Government Australian Fisheries Management Authority Corporate Plan 2023–2026 Including Annual Operational Plan 2023	‘Ensure the exploitation of fisheries and related activities is consistent with the principles of ecologically sustainable development’. (p. 6)
Australian Government Department of Agriculture, Fisheries and Forestry, Aqua Plan 2022–27: Improving aquatic animal health, profitability and productivity of aquatic animal industries and protecting aquatic environments	‘Improve the productivity and profitability of aquatic animal industries (including aquaculture, fisheries, and ornamental fish sectors) and to protect our unique aquatic environments from the threat of disease’. (Ministerial Introduction)
Australian Government Department of Agriculture and Water Resources Commonwealth Fisheries Bycatch Policy Framework for managing the risk of fishing-related impacts on bycatch species in Commonwealth fisheries, 2018	‘The Australian Government has revised the first Bycatch Policy with a commitment to a sustainable, productive, internationally competitive and profitable fisheries industry. The policy focuses on managing the direct and indirect impacts of fishing activities on the marine environment’. (Ministerial Introduction)
Australian Government Commonwealth Fisheries Harvest Strategy Policy Harvest Strategy Policy Framework for applying an evidence-based approach to setting harvest levels in Commonwealth fisheries, 2018	‘A further objective of the Fisheries Management Act 1991 is to pursue maximization of net economic returns to the Australian community from the management of fisheries’. (p. 4)
Australian Government Department of Agriculture, Fisheries and Forestry Corporate Plan 2023–24	‘Our objectives To achieve our purpose, we therefore focus on 3 objectives: 1) Industry growth—Support industry to grow towards a $100 billion agriculture, fisheries and forestry industry by 2030 amid changing global market conditions. 2) Biosecurity—Strengthen our national biosecurity system to provide an appropriate level of protection to Australia’s people, our environment and economy from the biosecurity threats of today and tomorrow. 3) Resilience and sustainability—Increase the contribution agriculture, fisheries and forestry make to a healthy, sustainable and low-emissions environment’. (pp. 3, 5, 6)
NSW Department of Primary Industries NSW Biosecurity and Food Safety Strategy 2022–2030	‘It has never been more critical to ensure we strategically invest in our biosecurity and food safety systems, not only as we seek to drive Australia’s *primary industries output to $100 billion ($30 billion in NSW) by 2030* but also as we confront a rapidly growing global threat profile’. (p. 2)
NT Department of Industry, Tourism and Trade Agribusiness 2030	‘Investment in biosecurity preparedness and prevention is the best kind of investment with the greatest returns to producers and the economy’. (p. 17)
Northern Territory Biosecurity Strategy 2016–2026	‘Plant and animal pests and diseases can threaten the present and future wealth of our primary production industries, with the potential to restrict or shut down business activity overnight’. Ministerial Forward
SA PIRSA & Government and Seafood Advisory Forum 2021–2031 Seafood Growth Strategy for South Australia	‘…the State Government and all stakeholders will partner through the Seafood Growth Strategy to realize the growth opportunities and potential for the South Australian seafood sector identified through the strategy’. (p. 9) And ‘Driving growth and development through new market opportunities, products and experiences’. (p. 12) And ‘Review and work to reduce regulatory framework to attract new investment and strengthen investment certainty’. (p. 15) ‘The Seafood Advisory Forum members agreed that refinement and review of the Act is required to ensure a more flexible management environment, increased industry efficiency and reduced costs for businesses’. (p. 20)
Growing Tasmanian Agriculture Research, Development and Extension for 2050	‘The Tasmanian Government has an ambitious goal to increase the annual value of the agricultural sector to $10 billion by 2050…. This White Paper was in part prompted by farmers and agribusinesses themselves who came to Government seeking to actively participate in developing strategies for achieving productivity improvements in agriculture to attain our shared growth targets’. (p. 13)
Tasmanian Department of Primary Industries, Parks, Water and Environment Tasmania’s Sustainable Agri-Food Plan 2019–23	‘The first Tasmanian Sustainable Agri-food Plan (the ‘Plan’), released in 2016, provided the impetus for growth and we are now delivering the updated new four-year Plan to ensure we remain on track to achieve our target farm gate value of $10 billion by 2050… The 2019–23 Plan continues this journey as we deliver on our AgriVision 2050 growth target and continue to create jobs and prosperity in rural communities across the state……’ (Ministerial introduction)
Queensland Department of Agriculture and Fisheries, Annual Report 2022–23	‘Sustainability Queensland’s agriculture, fisheries and forestry supply chains are changing to meet the state’s 2030 carbon emissions reduction target and lucrative market opportunities’. (p. 10)
WA Department of Primary Industries and Regional Development, 2022 Albany Aquaculture Development Zone Management Framework	‘Australia is well positioned to take advantage of the opportunity presented by the rapidly-increasing global demand for premium seafood due to our sustainable competitive advantages, which include the relatively pristine marine environment, professionally managed natural resources, reliable supply chain and geographical proximity to the rapidly-expanding Asian middle class’. (p. 2)
WA Department of Primary Industries and Regional Development Aquaculture Development Plan for Western Australia: Focusing on the key foundations for growth	‘Building community trust and support (‘social licence’) for sustainable aquaculture development is important for long-term industry success. This includes building both local and broader community support based on strong environmental and food production credentials’. (p. 11)

Victoria’s policies were an exception to the other states in that their suite of policies showed more focus on balance of health, sustainability and productivity needs. This was shown in a business plan (Victorian Fisheries Authority 2022) which set its vision as ‘to maintain healthy and sustainable fisheries for all Victorians’. The policy’s first focus area is ‘Healthy and Sustainable Fisheries’ and the first priority under this focus is ‘Victorian marine and freshwater fisheries, fish stocks and habitats are healthy and productive’. The Board of the Authority has a membership that is not dominated by industry representatives, whereas in other jurisdiction’s fisheries-related policies, when those involved in creating the policies are listed, government and industry representatives dominate. A Federal policy (Department of Agriculture Fisheries and Forestry 2022) exemplified that dominance where the national biosecurity strategic reference group had no environmental group representatives:

Australian Banana Growers’ Council, CSIRO, Freight and Trade Alliance, Invasive Species Council, National Farmers’ Federation, Seafood Industry Australia, Torres Strait Regional Authority, Rural Research and Development Corporations representative, Australian Pork Limited. (p. 3)

Although more encouragingly, the strategy does propose a future committee of diverse actors, including environmental and Indigenous stakeholders:

A National Biosecurity Strategy Implementation Committee (NIC) will be established, consisting of biosecurity stakeholders, including representatives from plant and animal industries, freight and logistics, aquatic industries, environmental groups, research organisations and Indigenous stakeholders. (p. 34)

Nevertheless, the current membership of that committee is still very heavily represented by industry (Australian Government 2025b). In South Australia, a seafood growth strategy (Government of South Australia 2021) was entirely concerned with growth and showed little consideration for the environment. The Strategy was devised by a Seafood Advisory Forum with members from industry, recreational and government sectors but contained no environmental voice. The strategy confidently stated:

In preparing for growth, it is essential that all seafood industry partners work under an agreed sustainability framework and that the impact of growth can be both accommodated and supported. (p. 16)

The ‘Fisheries Management Act 2007’ requires a South Australian policy concerning the abalone fisheries (Department of Primary Industries and Regions 2021) including a management plan to identify and assess (i) Current known impacts of the fishery on the ecosystem, (ii) Potential impacts of the fishery on the ecosystem, and (iii) Ecological factors that could have an impact on the performance of the fishery. Surprisingly, the policy contains no reference to HABs. We also found that the South Australian water security policy (Government of South Australia 2022) did not pay attention to the risks of runoffs from agriculture activity, which have been named as a contributing factor to the HAB. The policy shows strong concern with the economic necessity of water security but does not pay any attention to the impacts of water security on the marine environment.

The other notable feature of the agricultural and fishery policies is that, while they note the risks of climate change, they are largely silent in terms of calling for actions on climate change. Furthermore, they do not acknowledge Australia’s contribution to climate change as a major producer of fossil fuels and disproportionally high per capita emissions of greenhouse gases.

### Consideration of human health impacts

Overall, we found that the environment sector policies gave more consideration to human health than those in the agriculture sector. Given the role of agriculture in feeding the human population, it is surprising that the agricultural sector does not prioritize human health.

In the agriculture policies analysed, there was little linking of the identified biosecurity threats to human health impacts. While these would be the primary responsibility of the relevant Departments of Health, we found limited evidence of any linking mechanism or consideration of the human health impacts of HABs. Similarly, the threat of climate change was noted in multiple agriculture policies but there were very few statements about the need to reduce greenhouse gas emissions. The few that were there related to reducing emissions in the agriculture sector itself but made no reference to the importance of overall reduction.

We also looked for evidence of collaborative action across sectors which may have provided a framework for multisector responses to HABs but found very little evidence of such collaboration. Some policies mentioned the One Health approach which is defined by WHO (World Health Organization 2025) as ‘integrated, unifying approach that aims to sustainably balance and optimise the health of people, animals and ecosystems’, but this framing was rarely used to link agriculture issues to human health. For example, a NSW policy (Department of Primary Industries 2022) conceptualized the concept as linking to ‘prosperity’:

Recognising the relationships between animal, plant, environmental, and human health, this Strategy draws on the concept of ‘One Health’ and the interdependencies between *optimal biosecurity outcomes, food safety, and economic, social, and environmental prosperity*. (p. 4)

This conceptualization meant the implications of biosecurity issues such as a HAB for human health were not developed at all. The Queensland Department of Agriculture and Fisheries Annual Report 2022–23 did not explicitly mention the term ‘One Health’, but several initiatives and strategic actions within the report reflect core One Health principles, which emphasize the interconnectedness of human, animal, and environmental health. For example, it was stated:

…the objective is to mitigate the risks and impacts of animal and plant pests and diseases and weeds to the economy, the environment, social amenity and human health. (p. 79)

The Northern Territory approach was definitely not rooted in a One Health approach as its Biosecurity Strategy (2016–2026) (Northern Territory Department of Agriculture and Fisheries 2016) stated:

With the exception of zoonoses, the strategy will not address communicable diseases, as human biosecurity is a specific area of health, measures for which are outside the scope of this Strategy. (p. 9)

Given the current HAB is in South Australian waters we paid particular attention to that State’s policies. The Environmental Protection Agency of South Australia did include consideration of human health as one of the five focus areas on its Strategic Directions document:

Strengthening our position as a safe and great place to live and do business, with a sustainable lifestyle, and effectively utilising an environmental regulatory framework that provides for the wellbeing, health and safety of our people and community. (Environment Protection Authority 2022) (p. 4)

The document also noted that it:

provides a framework for leading the way in preventing and reducing harm, achieving better outcomes, and responding to the environmental challenges facing South Australia today and into the future. (p. 3)

In addition, the document speaks of how the government will respond to ‘environmental pressures’ and notes that they ‘will work closely with communities, industry, research bodies and governments to look for innovative ways to best manage environmental pressures and proactively identify emerging challenges and opportunities’. Taking the South Australian policies overall, there is a considerable concern for the impact of climate change on the population, but this is primarily in terms of impacts on landscapes and especially the projections for a drying climate and the need for water security. There was evidence of co-ordination with health. A key South Australian plan (Department for Environment and Water 2022) notes that it ‘was developed following consultation with South Australia’s emergency management, health, infrastructure, primary production and natural resources sectors’. (p. 1). Later on, the plan states: ‘Managing, conserving, and sustaining our natural environment is vital for the wellbeing of all South Australians and our economy’. (p. 16) but attention to the risks of HABs is missing.

## Discussion

Our review of the agriculture, fisheries, and environment policies of the Australian Federal, State, and Territory governments indicates a lack of consideration, and so preparedness, for the HAB that has been persistent in South Australia since March 2025 and no attention to the human health impacts. In this discussion we consider the possible reasons for this together with the implications for future policy making and preparedness.

### Anticipation of environmental crises

We found that there was largely a silence on the likelihood of such a HAB apart from in the state of Victoria. This was the case even in biosecurity policies where there was scant mention. The framing of the agriculture and fisheries policies was primarily about economic growth and the need to protect export industries. A concern with ecological sustainability was evident in the policies but in most cases framed this as a contributor to economic objectives. Concern with climate change was also evident and calls for lower-carbon practices were made but we found no calls for the country to reduce its mining, use and export of fossil fuels. The overall economic orientation of these policies indicates a meta-institutional orientation that reflects governments’ neo-liberal economic policy orientations. (Howlett *et al*. 2009). Our policy analysis indicates the ways in which such an orientation can become self-defeating when it does not account for the realities of climate change and environmental impacts. The impacts on the South Australian economy of the HAB particularly through impacts on fishing (Cox and Close-Brown 2025) and tourism (Lim 2025) are estimated to be substantive and increasing the longer the HAB persists.

The response to the South Australian HAB has been criticized as being slow (Kelsall 2025) and there was no clear mechanism to link Federal and State responses. The policies we reviewed did not indicate the presence or intention to establish mechanisms for rapid responses. While a $28 million funding package was agreed ($14 million federal support for clean-up, research, industry, and community relief and $14 million from South Australia for clean-up, community and industry support), it is likely to prove inadequate. Multiple submissions to the Senate Inquiry into the SA HAB noted the inadequacy of the funding packages (Senate Standing Committees on Environment and Communications 2025a, 2025b) (Chapter 3). The State government is also monitoring water conditions, issuing health and safety guidance and engaging the community—including weekly assessments by the State’s HAB taskforce and regular community forums (Government of South Australia 2025a). The unpredictability and severity of the SA HAB poses political threats for the incumbent governments especially as state election is due in March 2026 (Kelsall 2025).

In nearly all Australian jurisdictions there are policies that are directed to climate change, but these are primarily focused on terrestrial concerns and there is much less attention given to marine issues especially in the States and Territories. Federally there is more attention and that has intensified recently (Department of Climate Change Energy the Environment and Water 2025). Our findings indicate the need for increased attention to the marine environment in climate change policies. There are likely to be multiple ecosystem breakdowns Australia-wide of the type currently being experienced in South Australia. Indeed Hull (2021), writing in relation to the recurring HAB in Florida, notes the direct relationship to global warming induced ocean warming, acidification, and altered hydrolytic cycles. He notes that unless greenhouse gas emissions are curbed, ‘the likelihood of severe, pervasive, and irreversible impacts for people and ecosystems will increase’ (p. 116). Consequently, public policy needs to be significantly better prepared for their eventuality and also aware of the consequences of not acting on global warming related events that affect health and are going to be more common in the future.

### Lack of intersectoral perspectives in policies

The HAB is having significant physical and mental health impact across South Australia. Government advice notes skin and eye irritation, and respiratory symptoms including cough and shortness of breath through exposure to sea spray or aerosols (Government of South Australia 2025b). The Senate Standing Committees on Environment and Communications (2025a, 2025b) Inquiry submissions describe both physical and mental health effects, including distress from business losses and restricted access to the marine environment, a key source of wellbeing. Media reports have also pointed to the mental health impact (LeBusque 2025). Increasing evidence points to the existence of ‘solastalgia’ which is distress caused by detrimental environmental changes (Albrecht *et al*. 2007) and the Senate Inquiry submissions indicate this distress is widespread. These health impacts increase the need for effective intersectoral policies between health and other agencies responsible for aspects of environmental management. Cross-sectors planning for environmental harms are vital. Australia recently published its first National Climate Risk Assessment (NCRA) and National Adaptation Plan (NAP) (Australian Government 2025a). These documents do not explicitly mention HABs. However, they warn of the increased risk of climate tipping points and that ‘as climate hazards change in frequency and increase in severity, we are likely to experience more compounding, cascading and concurrent hazards, with greater potential impacts on Australia’s health, infrastructure, environment and economy’ (p. 5). It is possible that the HAB in South Australia is a tipping point as it shows no signs of disappearing and is projected to be present for some time.

Such tipping points expose the inadequacy of siloed policy responses. The SA HAB poses a wicked problem for public policymakers, lacking known short-term solutions. Public pressure for rapid action contrast with limited short-term options and long-term economic goals and constrains. This underlines the need for government policies across all sectors to align and prepare the country for complex ecological crises. These lessons have been recognized in health promotion ever since the WHO Alma Ata Declaration (World Health Organization 1978), the WHO Ottawa Charter’s (World Health Organization 1986) mandate on health public policy and more recent Health in All Policies initiative (World Health Organization 2023), but still have not been adopted in mainstream policy making.

### Bring voices for nature and the environment into policy making

A notable absence in the policies we reviewed was any voice for nature. Overwhelmingly, environmental protection was consistently framed through economic priorities, concern for nature rarely translated into strategic action. In Australian health promotion policy a ‘lifestyle’ drift away from structural change to a focus on behaviour change has been noted (Fisher *et al*. 2016). We see a similar ‘economic drift’ in Australian agriculture and environmental policy away from the rhetoric about the need to protect the environment in background statements to an absence of specific action for nature and on climate change to an emphasis on measures to support the economy. A review of Australia’s biosecurity policy noted that during the consultation for the review ‘multiple stakeholders (government, agriculture/aquaculture industries and environmental groups) commented that environmental biosecurity was undervalued in comparison to agricultural biosecurity’ (Allen 2025) (p. 36). The HAB demonstrates how neglecting nature’s protection directly undermines human health. Preserving marine environments is essential for current and future wellbeing.

In the broader policy landscape there is clear policy incoherence whereby, despite the evidence that HABs are caused in large part by the warming of the ocean, Australia continues to exploit its fossil fuel assets despite the very considerable climate risks it faces (Moon 2024). Many of the HAB Senate Inquiry (Senate Standing Committees on Environment and Communications 2025a, 2025b) submissions point to this incoherence. South Australian communities affected by the HAB have called for stronger action to fossil fuels extraction (Cox and Canales 2025).

### Harmful Algal Blooms, health, and policy in the future

The public policies we reviewed lack clear national and state pathways for co-ordinated HAB response, underscore the need to strengthen intersectoral co-ordination mechanism within and between government (World Health Organization 2023). In addition, more needs to be known about the long-term health effects of human interactions with HABs. A systematic scoping review (Young *et al*. 2020) concluded that: ‘Significant gaps in the evidence base for the association between marine HABs [harmful algal blooms] and human health have been demonstrated in this review. Notably, there is a lack of studies of chronic exposure….…’.

The lack of any conclusive evidence suggests the need for well-designed rigorous long-term studies to examine the HAB related health effects. As HABs increase globally and pose complex and difficult to solve problems (Anderson 2014, Lim *et al*. 2023), greater attention is needed to mitigation and adaptation strategies.

Another major challenge for governments in a world threatened by recurring ‘natural’ disasters is to change the narrative to ‘climate-induced’ disaster and to reframe policy so that it focuses more directly on the need to protect and value nature and much less on the short-term needs of the economy and business interests. The analysis of why this reframing is important has been laid out in Australia’s first NCRA (Australian Government National Climate Service 2025) and its companion NAP (Department Climate Change Energy the Environment and Water 2025) These documents will shape future climate governance and detail sixty-three nationally significant risks, mapped across eight systems. They paint a worrying picture, warning of escalating risks to homes, infrastructure, food, security, and health. One of the eleven risk systems analysed in detail is ‘Natural ecosystems—risks to ecosystems, landscapes and seascapes, including risk of ecosystem transformation or collapse, and loss of nature’s benefits to people’. This describes exactly what has occurred in the South Australian system, but the risk of the ecological collapse seen in South Australia because of the HAB was not canvassed in the report specifically. The NCRA and NAP provide a solid framework which really recognizes the risks of climate change across society. Effective implementation across sectors could encourage and enhance preparedness to respond to future crises like the HAB, but only if supported by more ambitious emissions targets.

### Limitations

Our study was based on analysis of agriculture and environmental policies that were current in 2024–25. Some policies had been in place for a decade and so collectively they were able to give us a picture of Australia’s preparedness for ecological crises, such as the HAB, but we acknowledge that written policy is only one aspect of government responses.

## Conclusion

We have reviewed Australian food-related agriculture and environment policies to determine the extent to which Australia is prepared to respond to ecological crises such as the South Australian HAB. Out of 180 policies, only 9 referenced HABs and this was done within an economic context rather than within human and environmental health framing. The South Australian case study provides a cautionary tale for other jurisdictions worldwide as ecological disasters are predicted to be more common in the future and will have severe direct and indirect impacts on human health throughout the world. Public policies need to be better prepared to anticipate and respond to these disasters. Adopting health promotion frames especially that of intersectoral healthy public policy will be a valuable tool in disaster response in the future.

## Supplementary Material

daaf240_Supplementary_Data

## Data Availability

All policies analysed were publicly available on websites at the time of collection (2024–25) and are listed in the Supplementary material.
