# Supplementary material for: Did Australian policy prepare for a harmful algal bloom with significant human health impacts? Analysis and lessons from South Australia
Source: Health Promot Int. 2026 Jan 21;41(1):daaf240. doi: 10.1093/heapro/daaf240 (PMC12822599; doi:10.1093/heapro/daaf240)
Supplement: daaf240_Supplementary_Data [file daaf240_supplementary_data.docx]

**Supplementary File: Policies included in the detailed analysis**

| **Mention of keywords (text search)** | **Fish** | **Aquaculture** | **Algal**  **Algae** |
| --- | --- | --- | --- |
| 1. Australian Government Australian Fisheries Management Authority Australian Fisheries National Compliance Strategy 2022–2026 |  | 2 |  |
| 1. Australian Government Australian Fisheries Management Authority Corporate Plan 2023–2026 Including Annual Operational Plan 2023 | 4 | 1 |  |
| 1. Australian Government Australia Fisheries Management Authority Annual Report 2022-23 | 80 | 5 |  |
| 1. Australian Government Australian Fisheries Management Authority International Compliance and Engagement Program 2022–24 | 14 | 2 |  |
| 1. Government AFMA position statement: How AFMA considers the social aspects of the ecologically sustainable development principles in the management of Commonwealth Fisheries (March 2019) | 1 | 1 |  |
| 1. Australian Government Australian Fisheries Management Authority AFMA Strategic Research Plan 2023-28 | 10 | 1 |  |
| 1. Dept. Climate Change, Energy, Environment and Water Annual Climate Change Statement 2023 | 1 |  |  |
| 1. Australian Government Department of Agriculture, Fisheries and Forestry, Aqua Plan 2022-27: Improving aquatic animal health, profitability and productivity of aquatic animal industries and protecting aquatic environments | 34 | 59 |  |
| 1. Australian Government Department of Agriculture, Fisheries and Forestry, Aqua Plan 2022-27 Communication and engagement plan | 1 | 1 |  |
| 1. Australian Government Department of Agriculture, Fisheries and Forestry, Aqua Plan 2022-27 Implementation plan | 1 | 1 |  |
| 1. Australian Government Department of Agriculture and Water Resources Australian Pest Animal Strategy 2017–2027   Invasive Plants and Animals Committee | 4 | 3 |  |
| 1. Australian Government Department of Agriculture Australia's Second National Plan of Action to   Prevent, Deter and Eliminate Illegal, Unreported and Unregulated Fishing, 2014 | 32 | 3 |  |
| 1. Australian Government DAFF Biosecurity 2030 Action Plan 2023: Update on progress and 2023 priority actions |  | 1 |  |
| 1. AG Department of Agriculture and Water Resources Commonwealth Fisheries Bycatch Policy Framework for managing the risk of fishing-related impacts on bycatch species in Commonwealth fisheries, 2018 | 24 |  |  |
| 1. AG Commonwealth Fisheries Harvest Strategy Policy Harvest Strategy Policy Framework for applying an evidence-based approach to setting harvest levels in Commonwealth fisheries, 2018 | 31 |  |  |
| 1. AG Department of Agriculture, Fisheries and Forestry Corporate Plan 2-23-24 | 3 |  |  |
| 1. AG Dept, Agriculture Water and the Environment Delivering Ag2030, April 2022 | 1 | 2 |  |
| 1. Australian Government Dept. Environment and water resources guidelines for the ecologically sustainable management of fisheries - 2007 | 8 |  |  |
| 1. AG Dept, Agriculture Water and the Environment National Plastics Plan 2021 | 1 |  |  |
| 1. AG Department of Agriculture and Water Resource National Aquaculture Strategy, 2017 | 18 | 292 | 1 |
| 1. AG DAFF National Biosecurity Strategy 2022-32 |  | 2 |  |
| 1. AG Department of Agriculture, Water and the Environment, National Soil Strategy, 2021 | 1 |  |  |
| 1. NSW Department of Regional Annual Report 2022 - 2023 | 24 | 12 |  |
| 1. NSW Department of Primary Industries NSW Biosecurity and Food Safety Strategy 2022 – 2030 | 4 | 1 |  |
| 1. NSW Department of Primary Industries Climate Change Research Strategy: Climate Vulnerability Assessment Methodology Report | 1 |  |  |
| 1. NSW Government Food Safety Strategy 2015 – 2021 Safe Food from Paddock to Plate | 4 | 3 |  |
| 1. NSW Dept Primary Industries Stronger Primary Industries Strategy, 2022– 20 | 10 |  |  |
| 1. NSW Food Authority Annual Report 2021–22 |  | 3 | 3 |
| 1. Deloittes for NSW Dept. Primary Industries NSW Agribusiness Positioned for prosperity Deloitte Access Economics report for the NSW Department of Primary Industries July 2016 | 3 | 13 |  |
| 1. NT Department of Industry, Tourism and Trade Agribusiness 2030 | 10 | 63 |  |
| 1. Northern Territory Biosecurity Strategy 2016-2026 | 2 | 2 |  |
| 1. Queensland Department of Agriculture and Fisheries Annual Report 2022–23 | 11 | 15 |  |
| 1. Queensland Sustainable Fisheries Strategy2017-27 | ? | 1 |  |
| 1. Queensland Department of Agriculture and Fisheries Strategic Plan 2023–2027 | 1 |  |  |
| 1. Queensland AgTech Roadmap 2023-28 |  | 4 |  |
| 1. Pathways to a climate resilient Queensland Queensland Climate Adaptation Strategy 2017–2030 |  | 1 |  |
| 1. SA CLIMATE CHANGE ACTIONS, 2022 |  | 2 |  |
| 1. South Australian Department of Environment and Water Climate change science and knowledge plan for South Australia \| 2022 Ensuring an evidence base to support South Australia’s response to climate change | 3 | 1 |  |
| 1. South Australian Department of Environment and Water State Landscape Strategy, 2021 | 1 | 1 |  |
| 1. SA DEW Water Security Statement 2022 Water for Sustainable Growth | 4 | 1 |  |
| 1. Department of Primary Industries and Regions Management Plan for the South Australian Commercial Abalone Fisheries (2021) | 12 | 9 |  |
| 1. SA PIRSA & Government and Seafood Advisory Forum 2021-2031 Seafood Growth Strategy for South Australia | 8 | 28 |  |
| 1. Tasmanian Government Tasmanian Salmon Industry Plan 2023 | 4 | 37 |  |
| 1. Growing Tasmanian Agriculture Research, Development and Extension for 2050 |  | 1 |  |
| 1. Tasmania’s Climate Change Action Plan 2023-25 June 2023 |  | 2 |  |
| 1. Tasmanian Department of Primary Industries, Parks, Water and Environment Tasmania’s sustainable agri-food plan 2019-23 | 1 | 5 |  |
| 1. Victorian Department of Environment, Land, Water and Planning (DELWP) l Building Victoria's Climate Resilience | 5 | 2 |  |
| 1. Victorian Department of Energy, Environment and Climate Action (DEECA) Corporate Plan 2023-2027 | 2 |  |  |
| 1. Victorian Department of Jobs, Skills, Industry and Regions Annual Report 2022-23 | 9 | 2 |  |
| 1. Victoria Department of Jobs, Precincts and Regions Primary Production Climate Change Adaptation Action Plan 2022–2026 | 11 |  |  |
| 1. Victorian Fisheries Authority 2019-2024 Strategic Plan | 2 |  |  |
| 1. Victorian Fisheries Authority Business Plan 2022-23 | 42 | 25 |  |
| 1. Department of Environment, Land, Water and Planning Victorian Climate Change Strategy, 2021 | 1 |  |  |
| 1. Department of Jobs, Precincts and Regions 2022 Victorian Primary Production Climate Change Adaptation Action Plan 2022–2026 |  |  |  |
| 1. Victorian Department of Environment, Land, Water and Planning Water Cycle Climate Change Adaptation Action Plan 2022–2026 | 1 |  |  |
| 1. WA Department of Primary Industries and Regional Development, 2022 Albany Aquaculture Development Zone Management Framework | 25 | 289 | 4 |
| 1. WA Department of Primary Industries and Regional Development Aquaculture Development Plan for Western Australia: Focusing on the key foundations for growth | 35 | 227 |  |
| 1. WA DPIRD Aquaculture of Seaweed in Western Australia, 2022 |  | 79 |  |
| 1. WA Department of Water and Environmental Regulation Climate Adaptation Strategy Building WA’s climate resilient future July 2023 | 5 |  |  |
| 1. WA Department of Primary Industries and Regional Development Annual Report 2022-23 | 71 | 37 | 3 |
| 1. WA Department of Water and Environmental Regulation Annual report 2022–23 | 3 |  |  |
| 1. WA Department of Primary Industries and Regional Development Primary Industries Plan 2020–2024 | 4 | 23 |  |
| 1. WA Department of Primary Industries and Regional Development Western Australian Natural Resource Management Framework 2018 | 4 | 1 |  |
